# Correlation of Brain Neuropeptide (Nesfatin-1 and Orexin-A) Concentrations with Anthropometric and Biochemical Parameters in Malnourished Children

**DOI:** 10.4274/jcrpe.1930

**Published:** 2015-08-31

**Authors:** Feyza Ustabaş Kahraman, Aysel Vehapoğlu, İlker Tolga Özgen, Şule Terzioğlu, Yaşar Cesur, Ruşen Dündaröz

**Affiliations:** 1 Bezmialem Vakıf University Faculty of Medicine, Department of Pediatrics, İstanbul, Turkey; 2 Bezmialem Vakıf University Faculty of Medicine, Department of Pediatric Endocrinology, İstanbul, Turkey; 3 Bezmialem Vakıf University Faculty of Medicine, Department of Medicinal Biology, İstanbul, Turkey

**Keywords:** Underweight children, appetite regulation, nesfatin-1, orexin-A

## Abstract

**Objective:**

Malnutrition continues to be a leading cause of stunted growth in many countries. This study aimed to investigate serum nesfatin-1 and orexin-A levels in underweight children and the potential correlations of these levels with anthropometric and nutritional parameters.

**Methods:**

The study enrolled 44 prepubertal children (between 2 and 12 years of age) with thinness grades of 1-3 and 41 healthy age- and gender-matched children. The demographic, clinical and laboratory parameters including nesfatin-1 and orexin-A concentrations were compared between the two groups. The correlations of nesfatin-1 and orexin-A with biochemical and anthropometric parameters were investigated. The receiver operating characteristic (ROC) analysis were also performed for evaluating nesfatin-1 and orexin-A in distinguishing children with malnutrition from healthy controls.

**Results:**

Thyroid-stimulating hormone, vitamin B12 and insulin levels were significantly lower in the study group than controls (p=0.001, p=0.049 and p=0.033, respectively). Mean nesfatin-1 levels in the malnourished group was also significantly lower compared to the healthy controls (3871.2±1608.8 vs. 5515.0±3816.4 pg/mL, p=0.012). No significant difference was observed in the orexin-A levels between the two groups (malnourished vs. control groups: 1135.7±306.0 vs. 1025.7±361.6 pg/mL, p=0.141). Correlation analyses revealed a positive correlation of nesfatin-1 and a negative correlation of orexin-A with body mass index (BMI) z-score. ROC analysis demonstrated that nesfatin-1 and orexin-A cannot be used to distinguish children with malnutrition from healthy controls (AUC: 0.620, p=0.061 for nesfatin-1 and AUC: 0.584, p=0.190 for orexin-A).

**Conclusion:**

The positive correlation of nesfatin-1 and the negative correlation of orexin-A with BMI suggest that these neuropeptides may be a part of a protective mechanism in the maintenance of nutritional status and that they may have a role in regulating food intake in undernourished children.

## INTRODUCTION

The world is undergoing a nutritional transition with distinct characteristics and stages that exhibit a marked increase in the prevalence of overweight and obesity at different stages of life. Nevertheless, malnutrition is still a health problem in childhood and protein-energy malnutrition remains one of the most important health problems in developing countries. Children are especially threatened by malnutrition because of the high protein-energy cost of growth. In developed countries, reduced appetite and low food intake are primarily due to premature birth, chronic disease and anorexia nervosa. In the United States, a developed country, 3% of children are stunted, while in Egypt, a developing country, 29% of the children younger than 5 years are stunted and 11% are wasted. In Turkey, 12% of children are stunted and 1% are wasted (1). The prevalence of stunting and overweight/obesity was 9.1% and 24.0%, respectively, in Brazilian children from 5 to 19 years of age (2).

Nesfatin-1, a recently identified anorexigenic brain neuropeptide that regulates appetite, food intake and body weight, is a potent physiological inhibitor of food intake and body weight. Nesfatin-1 has the capacity to cross the blood-brain barrier and its signaling seems to occur independently of the leptin-melanocortin signaling pathway (3). Plasma nesfatin-1 levels were reported to be decreased in patients with type 2 diabetes mellitus (4). The relationship of nesfatin-1 with the body mass index (BMI), plasma insulin and homeostasis model assessment of insulin resistance (HOMA-IR) has also been demonstrated (5). Nesfatin-1 is expressed in the hypothalamic nuclei and brain stem. However, few data exist on nesfatin-1 secretion in malnourished children (6).

Orexins are expressed in the posterolateral hypothalamic area, which has been described as the “feeding center” and these peptides were initially characterized as orexigenic (appetite-stimulating) factors (7). Orexin-A is a 33-amino-acid peptide with an N-terminal pyroglutamyl residue and two intra-chain disulfide bonds. Orexin-A is identical in several mammalian species (humans, mice, rats, cows and pigs). Orexin neurons have connections with neuropeptide Y (NPY) and agouti-related peptide neurons in the arcuate nucleus (8). The anatomical location of orexin-expressing neurons and their interactions with orexigenic peptides suggest that they play an important role in controlling food intake. However, orexin not only regulates appetite, but also contributes to sleep/wakefulness, thermoregulation, energy homeostasis and reward systems (7). Orexin-A, when administered intracerebroventricularly, stimulates food intake and energy expenditure in a dose-dependent manner in young rats (8). Orexin-A can be determined in human plasma and its level might reflect the peptide coming from the brain. Orexin-A can also be produced in the neurons of the submucosal and intramuscular plexuses of the gastrointestinal tract in humans and rodents (8). Adam et al (9) found that orexin levels were diminished in obese subjects, while another study demonstrated that the orexin-A concentration was correlated negatively with BMI in normal subjects (10).

This present study aimed to investigate serum nesfatin-1 and orexin-A levels in childhood malnutrition and their potential correlations with food intake, anthropometric indices and body composition.

## METHODS

The study included 44 undernourished children and a control group of 41 healthy children. The study group consisted of prepubertal children above 2 years of age with thinness grades 1-3 and a current weight less than 90% of the ideal body weight (IBW). The study participants were recruited from Bezmialem Vakıf University Pediatric Clinic between March 2014 and July 2014. Children with chronic diseases (cardiovascular, gastrointestinal or respiratory), a history of drug use (steroids or antipsychotics), endocrine pathology (Cushing syndrome or hypothyroidism), or suspected syndromes were excluded from the study. Each participant underwent a detailed physical examination, including anthropometric measurements and estimation of the degree of malnutrition. Standing height was measured to the nearest 0.1 cm with a Harpenden fixed stadiometer. Body weight (kg) was measured on a Seca balance scale to the nearest 0.1 kg, with each subject dressed in light underwear. BMI was calculated by dividing the weight by height in meters squared (kg/m2). Thinness was classified as grades 1, 2 and 3 according to BMI z-scores cut-off values which were reported by Cole et al (11).

All blood analyses were performed on fasting samples in both the study and control groups. The red blood cell count and red blood cell distribution width were assessed on an ADVIA 2120 hematology analyzer (Siemens, Washington DC, USA). Serum ferritin was measured with an immunoassay using a Siemens ADVIA Centaur XP analyzer. Vitamin B12 was measured via electrochemiluminescence immunoassay (ECLIA). Glucose was measured using a glucose oxidase technique (Siemens ADVIA 1800) and the insulin levels were analyzed with a direct chemiluminescence technique (Siemens ADVIA Centaur XP). Insulin resistance was estimated from the fasting plasma measurements using the HOMA-IR [insulin (Mu/L)×glucose (mmol/L)/22.5]. The insulin resistance criteria were HOMA-IR >2.5 for prepubertal children and HOMA-IR >4.0 for adolescents (12). Thyroid hormone levels [free thyroxine (fT4), thyroid-stimulating hormone (TSH)] were measured using a direct chemiluminescence technique (Siemens ADVIA Centaur XP).

The blood samples were centrifuged for 10 min at 4500 rpm and 4 ˚C. The resulting serum samples were stored at -80 ˚C until assayed. The serum concentrations of human orexin-A (cat. no. CEA607Hu) and nesfatin-1 (cat. no. CEA242Hu) were determined using enzyme-linked immunosorbent assay (ELISA) kits from USCN Life Science (Wuhan, China), according to the manufacturer’s protocols. A Multiskan FC® microplate photometer (Thermo Fisher Scientific, Waltham, MA, USA) was used for reading the intensity of color at 450 nm. The concentrations were calculated based on standard curves and expressed in pg/mL. The detection ranges of the kits were 12.4-1000 pg/mL and 617.3-50000 pg/mL for orexin-A and nesfatin-1, respectively. The intra-assay variability of the both kits was less than 10%. Analyses with ELISA kit were performed once.

The study was approved by the local ethics committee. Written consent from the parents of each subject was also obtained before the study.

### Statistical Analysis

SPSS was used for the statistical analysis (ver. 17.0; SPSS Inc., Chicago, IL, USA). Numerical parameters are presented as means ± standard deviations and the distributions of categorical measurements are presented as frequencies and percentages. The subjects’ demographic and clinical characteristics were compared between the groups using independent t-tests. Relationships of Δ-differences were evaluated using Pearson’s correlation analysis and categorical data were compared with the chi-square test. A p-value <0.05 was accepted as indicating statistical significance. The receiver operating characteristic (ROC) analysis were also used for the assessment of the performances of orexin-A and nesfatin-1 to distinguish children with malnutrition from healthy controls.

## RESULTS

Age and gender distributions were not significantly different between the two groups. The mean age was 7.3±2.8 years for patients and 7.3±3.0 years for controls (p=0.937). The study group included 25 (56.9%) males and 19 (43.1%) females and the control group-24 (58.6%) males and 17 (41.4%) females. In the patient group, 21 (47.7%), 19 (43.2%) and 4 (9.1%) children were classified as thinness grades 1-3, respectively. No significant differences in HOMA-IR, serum albumin and ferritin levels, height and height z-scores, were observed between the patients and controls, while significant differences were detected in the weight, weight z-score, BMI, BMI z-score, and IBW%. The fT4 level was higher in the undernourished group. TSH, vitamin B12 and insulin levels were significantly lower in the study group than controls. The comparison of the demographic, clinical and laboratory features of the groups with p values are given in Table 1. Plasma nesfatin-1 level was also lower in the study group (p=0.012), while no difference was observed regarding the orexin-A levels (p=0.141).

We also examined the correlations of the plasma nesfatin-1 and orexin-A levels with clinical and laboratory parameters. No correlation was observed between nesfatin-1 and clinical and laboratory parameters except BMI z-score (r=0.219, p=0.048). In contrast to positive correlation of nesfatin-1, orexin-A was negatively correlated with BMI z-score (r=-0.246, p=0.026). The correlation coefficients and p-values are given in Table 2.

ROC analyses revealed that nesfatin-1 and orexin-A cannot be used to distinguish children with malnutrition from healthy controls (AUC=0.620, p=0.061 for nesfatin-1; AUC=0.584, p=0.190 for orexin-A).

## DISCUSSION

Despite improved economic conditions in Turkey in recent years, in our daily practice, many children are still being admitted to the hospital because of thinness. However, the nutritional status of these children is not always associated with the poor socioeconomic status of their family. The parents generally complain that their children have poor appetite. It is possible that the cause of undernutrition in these children may be related to a disorder in mechanisms regulating food intake. A communication between gastrointestinal tract, adipose tissue and brain satiety center is required for controlling food intake. A variety of peptides and hormones are involved in the pathophysiology of this communication (13). Nesfatin-1 is one of those peptides that is also thought to be involved in the physiological regulation of feeding behavior and body weight by suppressing food intake and peristalsis (14). Nesfatin-1 is an anorexigenic neuropeptide. Mortazavi et al (15) have demonstrated that chronic peripheral administration of nesfatin-1 reduces food intake and modulates whole body energy homeostasis. Abaci et al (16) have reported low nesfatin-1 levels in obese children and this finding has suggested that deficiency in nesfatin-1 may cause obesity because of increased appetite. A negative correlation between nesfatin-1 and BMI in non-obese male has also been shown (17). Based on the results of these studies, one of the hypotheses of our study was that high nesfatin-1 levels may lead to lack of appetite and consequently, to undernutrition in children. However, in contrast to our expectations, low nesfatin-1 levels were found in our study. In a recent study, Ogiso et al (6) have also reported low nesfatin-1 levels in patients with anorexia nervosa, a finding similar to our results. In this study, the authors have suggested that nesfatin-1 levels are regulated by nutritional status and that they respond to starvation. Our findings also suggest that low nesfatin-1 levels may be a preventive response to protect the organism against worsening nutritional status.

Another hypothalamic neuropeptide, orexin-A, is also involved in the regulation of food intake and the sleep/wake cycle (18). It has been shown that the cerebroventricular administration of orexin-A increases food intake in rodents and zebrafish (19,20). Moreover, an increase in the concentration of orexins in the cerebrospinal fluid during food deprivation has been observed in rats (21). Tomasik et al (10) found a negative correlation between orexin-A and BMI and a positive correlation with caloric intake in children with normal nutritional status. These authors have concluded that the plasma concentrations of orexin A and orexin B mimic the caloric demand and that orexins play a significant role in the regulation of energy balance in children. In contrast, Matsumura et al (22) have demonstrated that the orexin-A concentration was positively correlated with BMI in subjects with chronic obstructive pulmonary diseases (COPD). These authors have explained this paradoxical phenomenon by the role which orexin-A plays not only in the promotion of body weight change but also in the regulation of energy metabolism. They have stated that what happens nutritionally in patients with COPD is quite different from that which occurs in subjects who are merely depleted. We also found a negative correlation between plasma orexin-A levels and BMI z-scores.

The modifying effect of methyl group vitamins including vitamin B12 on expression of the obesogenic phenotypes and hypothalamic food intake regulatory systems in the Wistar rat offspring has been demonstrated (23). The triiodothyronine (T3) is known to play an important role in energy balance. Hypothalamus, a key area of the brain involved in energy homeostasis and regulation of food intake, is a target for T3. However, the components of these mechanisms are not yet well understood (24). Tohma et al (25) have reported that the orexin-A and nesfatin-1 levels are markedly affected by hyperthyroidism. Insulin also affects food intake regulation (26) and recently, a positive correlation between nesfatin-1 and HOMA-IR has been demonstrated (5). Furthermore, orexin-A contributes to the central regulation of glucose homeostasis (27). In addition to the effects of nesfatin-1 on metabolic regulation and food intake, animal studies have also suggested that nesfatin-1 enhances insulin release and leads to a time-, dose- and insulin-dependent reduction in blood glucose. These results suggest that nesfatin-1 takes part in the regulation of both glycometabolism and thyroid hormone functions (4). Therefore, we also investigated the relationship of nesfatin-1 and orexin-A with vitamin B12, fT4, TSH and HOMA-IR in undernourished children. However, no correlation was observed between the investigated parameters in our study group.

Several anthropometric indices have been used for many years to estimate the prevalence of thinness among children. Weight for height is a commonly used measurement of nutritional status and has been used as a measure of acute malnutrition. It is calculated as: percent of IBW (%)=[(weight of patient)/(weight of a normal child of the same height)]×100} (28). In recent years, a consensus has been growing that sex- and age-specific BMI percentiles be used as cut-off values for assessing overweight and obesity, as well as thinness/underweight in children older than 2 years. Therefore, we classified thinness according to the international cut-offs for BMI as thinness grades 1, 2 and 3 by sex for exact ages between 2 and 18 years, as defined by Cole et al (11). These constitute the clinical parameters to define malnutrition. On the other hand, the observed correlations between BMI, nesfatin-1 and orexin-A and moreover, previous comments about the possible ability of nesfatin-1 and orexin-A to mimic the caloric demands and nutritional status suggest that these two peptides may be used as laboratory markers to determine nutritional status. Therefore, we performed ROC analyses. However, our results revealed that nesfatin-1 and orexin-A cannot be used to distinguish children with malnutrition from healthy controls.

In conclusion, in this study, BMI z-scores were correlated negatively with orexin-A and positively with nesfatin-1 levels in children with malnutrition. Our results suggest that the decrease in nesfatin-1 and the increase in orexin-A levels with diminishing BMI z-score may be a part of compensatory mechanism against worsening weight loss in undernourished children.

## Figures and Tables

**Table 1:**
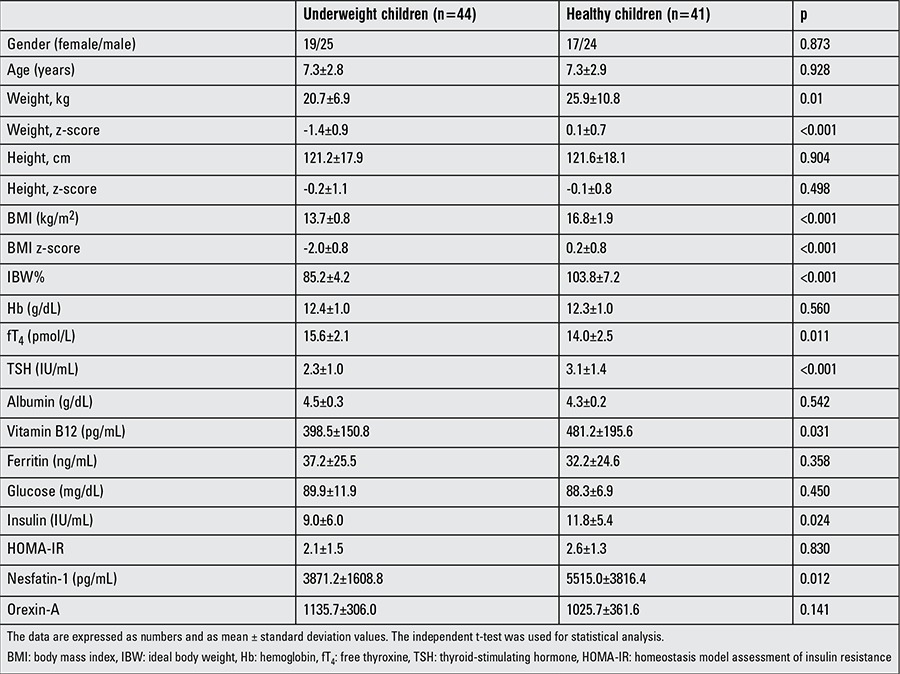
Demographic and laboratory characteristics of underweight children and controls

**Table 2:**
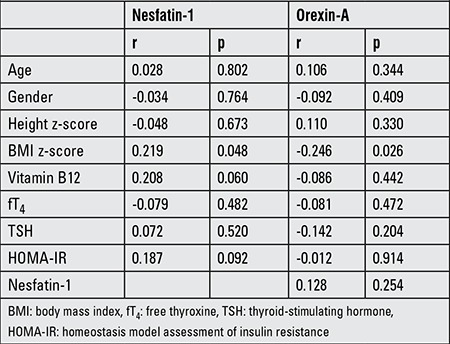
Bivariate correlations of nesfatin-1 and orexin-A with clinical and laboratory parameters (coefficients and p-values)
